# Dr. W. H. Dwinelle

**Published:** 1896-03

**Authors:** 


### DR. W. H. DWINELLE.

In the death of Dr. Dwinelle, which took place at Cazenovia, New York, February 13, 1896, the dental profession has lost one of its most prominent leaders. If dental literature be examined for the past fifty years, few names will appear more frequently and almost always connected with an original idea. In fact, many of the so-called new things in practice will be found to have had their origin in his prolific brain. In a period of selfish exclusiveness he was generous with his original ideas, giving these as freely as he was open-handed and open-hearted in other directions.

Dentistry should reverently hold in memory such a man, for truly there will not soon be seen again one more worthy of high regard.

He lived beyond the period usually allotted to humanity, and his co-workers can part with him, as we must part with all, conscious that the reputation he made and the good he did in his life will live as long as men read and appreciate the work of the fathers.

A full account of his life and work will appear in the next number.

